# Neuroimaging and Neuropathologic Findings in Pediatric Patients with ECMO: An Imaging–Pathology Comparison

**DOI:** 10.1007/s12028-026-02545-9

**Published:** 2026-05-26

**Authors:** Margherita Tabet, Bill Zhang, Chasity Custer, Michael Craig Morriss, Sumit Singh, Aliya Abioye, Priscilla Yu, Ethan Sanford, Lakshmi Raman, Veena Rajaram, David R. Busch

**Affiliations:** 1 Department of Anesthesiology and Pain Management, UT Southwestern Medical Center, Dallas, TX USA; 2 Division of Pediatric Critical Care Medicine, UT Southwestern Medical Center/Children’s Health, Dallas, TX USA; 3 Department of Radiology, UT Southwestern Medical Center/Children’s Health, Dallas, TX USA; 4 Division of Pediatric Cardiology, UT Southwestern Medical Center/Children’s Health, Dallas, TX USA; 5 Department of Pathology, UT Southwestern Medical Center/Children’s Health, Dallas, TX USA; 6 Department of Neurology, UT Southwestern Medical Center, Dallas, TX USA; 7 Department of Biomedical Engineering, UT Southwestern Medical Center, Dallas, TX USA

**Keywords:** Pediatrics, Brain injuries, Neuropathology, Neuroimaging, Extracorporeal membrane oxygenation, Critical care, Neurodiagnostic techniques

## Abstract

**Objective:**

Neurologic complications are a leading cause of morbidity and mortality in children supported with extracorporeal membrane oxygenation (ECMO). Antemortem neuroimaging is limited, while postmortem neuropathology provides definitive but rarely available assessment. Correlating imaging with pathology may improve diagnostic accuracy and monitoring strategies.

**Design:**

We performed a retrospective, single-center cohort study of neonates and children (0–18 years) supported with ECMO (2009–2024) who underwent cerebral autopsy. Standardized neuropathologic sampling across six brain regions classified injuries as mild, moderate, or severe. Antemortem imaging (computed tomography [CT] or head ultrasound [HUS]) within 7 days of death was reviewed by a blinded neuroradiologist and scored using a Neurologic Injury Severity (NIS) framework. Imaging–pathology correlation was assessed in patients with both datasets.

**Measurements and Main Results:**

A total of 54 patients underwent neuropathologic evaluation; 26 had qualifying neuroimaging. Neuropathology revealed frequent ischemic and hemorrhagic injuries, most commonly in the frontal lobes, deep grey matter, and temporal lobes. Severe injury was more frequent in venoarterial ECMO and in infants/toddlers. NIS scores strongly correlated with global and regional histopathologic severity, particularly in frontal, temporal, and deep grey regions. Discordance was observed in parieto-occipital, pontine, and cerebellar regions, where immunohistochemistry (GFAP, CD68) detected subtle injuries not visible with hematoxylin and eosin (H&E). Severe pathology consistently corresponded to NIS ≥ 10, while mild pathology aligned with low scores and limited involvement.

**Conclusions:**

Structured imaging severity scoring, especially with CT, correlates strongly with neuropathology in pediatric decedents of ECMO. Findings support NIS scoring as a surrogate for underlying pathology, while underscoring the need for refined histologic methods and adjunctive neuromonitoring to optimize neurologic surveillance.

**Supplementary Information:**

The online version contains supplementary material available at https://doi.org/10.1007/s12028-026-02545-9.

## Introduction

Extracorporeal membrane oxygenation (ECMO) is a life-support intervention for neonates and children with severe cardiac or respiratory failure, with more than 90,000 pediatric cases globally [1]. Pediatric survival has improved, now averaging around 54% across indications including extracorporeal cardiopulmonary resuscitation (ECPR), cardiac, and pulmonary support [2]. Despite these advances, neurologic complications remain a major cause of morbidity and mortality [3]. Acute brain injuries (ABIs), such as hypoxic-ischemic injury, ischemic stroke, and intracranial hemorrhage, are particularly devastating and often difficult to detect during ECMO [4]. Critically, in this high-acuity population, ECMO often serves as a marker of profound physiological instability rather than the sole catalyst for injury. This is especially relevant in ECPR cases, where significant hypoxic-ischemic insults often precede cannulation. Neurologic assessment is hindered by sedation, neuromuscular blockade, and the risks of patient transport. Routine neuroimaging is therefore reserved for high-suspicion scenarios such as pupillary abnormalities, new focal EEG findings, unexplained declines in hemoglobin, or acute neurologic deterioration [5–7]. In some cases, imaging informs discussions about withdrawal of life-sustaining therapies. The most common modalities are head ultrasound (HUS) and computed tomography (CT), while magnetic resonance imaging (MRI) remains rare due to technical and safety challenges [8]. Subtle or early injuries, however, are often missed [9]. Comprehensive postmortem neuropathologic assessments are also infrequent, limiting correlation between imaging and histopathology [10]. In adult ECMO, postmortem studies consistently reveal high ABI rates, especially hypoxic-ischemic injury linked to impaired autoregulation and altered perfusion in venoarterial ECMO (VA-ECMO) [11, 12]. Many of these injuries are underrecognized clinically [13]. Pediatric studies show similar patterns, with ischemic and hemorrhagic injuries exacerbated by the vulnerabilities of the immature brain [3]. However, it is often difficult to separate these neuropathologic findings from hypoxia and ischemia preceding ECMO without serial imaging and exams immediately following cannulation. Neuropathology provides critical insights, as histologic evaluation often reveals injuries undetected by imaging [14–17]. These discrepancies highlight the need for improved diagnostic strategies [18].

This study aims to characterize the types and distribution of neurologic injury in pediatric decedents of ECMO, compare antemortem neuroimaging findings with postmortem neuropathology, and identify diagnostic limitations relevant to neuromonitoring strategies.

## Methods

### Study Design and Oversight

This observational, single-center cohort study was conducted at Children’s Medical Center, Dallas, in accordance with the Committee for the Protection of Human Subjects at the University of Texas Southwestern Medical Center. The protocol (STU-2022–0723) was approved by the institutional review board on 12 September 2024. The institutional review board waived the requirement to obtain informed consent for research.

### Study Population

The patients were neonates and children (0–18 years) supported with ECMO between 1 January 2009 and 1 August 2024. Inclusion required death during ECMO or within 3 days of decannulation, with cerebral autopsy performed. For the imaging subanalysis, eligible patients also had neuroimaging within 7 days of death.

### Demographic and Clinical Data Collection

Demographic and baseline clinical data, including sex, race, ethnicity, height, weight, primary diagnosis, ECMO type (veno-venous [VV] or veno-arterial [VA]), oxygenator, and ECPR status, were abstracted from electronic health records and autopsy reports. For patients requiring ECPR, we recorded the duration of the cardiac arrest (minutes from the start of chest compressions to stable ECMO flow) and the specific cardiac or respiratory diagnosis at the time of the cardiac arrest to better characterize the pre-ECMO hypoxic burden. Continuous variables were reported as means ± standard deviation, and categorical variables as counts and percentages.

### Neuropathologic Examination

Postmortem examinations were performed by a board-certified neuropathologist blinded to imaging and clinical data. Gross examination was performed and sections taken for microscopic examinations with hematoxylin and eosin (H&E). When no obvious injury was observed, immunohistochemistry with GFAP and CD68 was performed to detect gliosis and microglial activation. Histological sampling was standardized and uniformly applied across all cases, including six brain regions: frontal lobes, deep grey matter, temporal lobes, parieto-occipital lobes, pons, and cerebellum. The sampling approach was informed by previously published neuropathologic studies in adult [19] and infant [20] ECMO populations and was implemented consistently across specimens. Histopathologic assessment was descriptive and was not based on predefined histopathologic criteria established a priori.

### Contextualized Classification of Neuropathologic Injury

Neuropathologic injuries were classified into mild, moderate, and severe categories based on histologic findings. Mild injury was defined as limited or nonspecific abnormalities, such as absent to minimal white matter injury, disorganized myelination, or slight ventricular dilation. These were usually scattered and restricted to one or two regions, without overt parenchymal damage. Moderate injury referred to more focal or localized abnormalities, such as white matter pallor, focal hemorrhage, ischemic changes with gliosis, or neuronal necrosis, often spanning multiple regions but not reflecting diffuse involvement. Severe injury encompassed widespread, multifocal pathology, including diffuse gliosis, multiple hemorrhages, subdural hemorrhage with necrosis, cortical edema, and necrotic infarcts. These findings often involved both cortical and subcortical regions, including the brainstem and cerebellum, and reflected diffuse cerebral injury. Each finding was mapped across brain regions, enabling structured quantification of anatomical burden (Electronic Supplemental Material [ESM] Table S1).

### Neuroimaging Acquisition, Analysis, and Neurologic Injury Severity (NIS) Score Determination

Antemortem imaging included CT and HUS within 7 days prior to death, with image quality sufficient for NIS scoring. When both were available, CT was prioritized for analysis due to its superior spatial resolution and diagnostic detail. All studies were reviewed by a neuroradiologist blinded to clinical and pathologic data. Intracranial injuries related to ECMO include both hemorrhagic and ischemic lesions, as well as increases in subarachnoid spaces and/or ventriculomegaly. Hence, a scoring methodology that incorporates all of these elements was done by Taylor et al. [21, 22] using head ultrasound and CT imaging for neonatal patients on ECMO. Using this score, they identified an inverse correlation between higher (worse) scores and psychomotor and mental developmental indices of the Bayley Scale [23]. We modified the category descriptions in the Taylor scoring system to add greater anatomic specificity, validating these modified scores using both CT and MRI in a wider age range of pediatric patients against Pediatric Cerebral Performance Category scales (PCPC). NIS categories were defined as mild (0–3), moderate (4–9), and severe (≥ 10).) Although PCPC was not directly measured in our study, prior ECMO cohorts have demonstrated an association between higher NIS and worse PCPC outcomes, with NIS scores ≥ 10 associated with poorer neurologic function [24]. Accordingly, the NIS severity categories were conceptually aligned with PCPC outcome ranges described in previous ECMO studies.

### Subanalysis: Neuropathology and Neuroimaging Correlation

Of the 54 patients with neuropathology, 26 had qualifying neuroimaging for correlation. For each, brain regions were graded (none, mild, moderate, severe) by histopathology and compared against NIS scores.

## Results

### Demographics

A total of 54 patients with ECMO underwent neuropathologic evaluation (29 males, 25 females). Most were White (70%) or Black (28%), and 72% were non-Hispanic. The mean weight was 20.8 kg, and mean height was 92.7 cm. Age groups included 9 neonates, 17 infants/toddlers, 15 children, and 13 adolescents. Pulmonary diagnoses accounted for 44% of cases, cardiac for 17%, and ECPR for 39%. Specifically, for the ECPR cohort, the median duration of cardiac arrest was 34 min (range 3–77 min), representing a significant period of pre-cannulation hypoxia–ischemia. Within this subgroup, 74% (17/23) of patients had underlying congenital heart disease, and 9% (2/23) had a documented genetic syndrome, such as DiGeorge syndrome. Notably, 70% (16/23) of these patients requiring ECPR had no clinically documented neurological complications while on ECMO prior to the imaging used for this study, suggesting that many injuries may have been established during the initial arrest or remained clinically silent during support. VA ECMO supported 56% of patients, while VV ECMO supported 44%. The most frequent oxygenator was the Quadrox i (56%) (Table 1). Notably, the vast majority of patients (83%) died from refractory cardiopulmonary failure rather than primary neurologic catastrophe.

**Table 1:** Demographic characteristics of the participants

Sample characteristics	Number (*n*)	Percentage (%)	Mean (μ)	Standard deviation (σ)	Range
Sex					
Male	29	53.7			
Female	25	46.3			
Race					
White or Caucasian	38	70.4			
Black	15	27.8			
Other/unknown	1	1.8			
Ethnicity					
Hispanic or Latino	15	27.8			
Not Hispanic or Latino	39	72.2			
Age category					
Adolescents	13	24.1			12–18 years
Children	15	27.8			4–11 years
Infants and toddlers	17	31.5			29 days–3 years
Neonates	9	16.6			0–28 days
Primary diagnoses					
Pulmonary	24	44.4			
ECPR	21	38.9			
Cardiac	9	16.7			
Type of ECMO					
VA ECMO	30	55.6			
VV ECMO	24	44.4			
Continuous variables					
Weight (kg)			20.79	22.63	2.3–98.2
Height (cm)			92.69	43.29	44.0–180.0

### Correlation of Gross and Microscopic Neuropathologic Findings by Brain Region

**Fig. 1:**
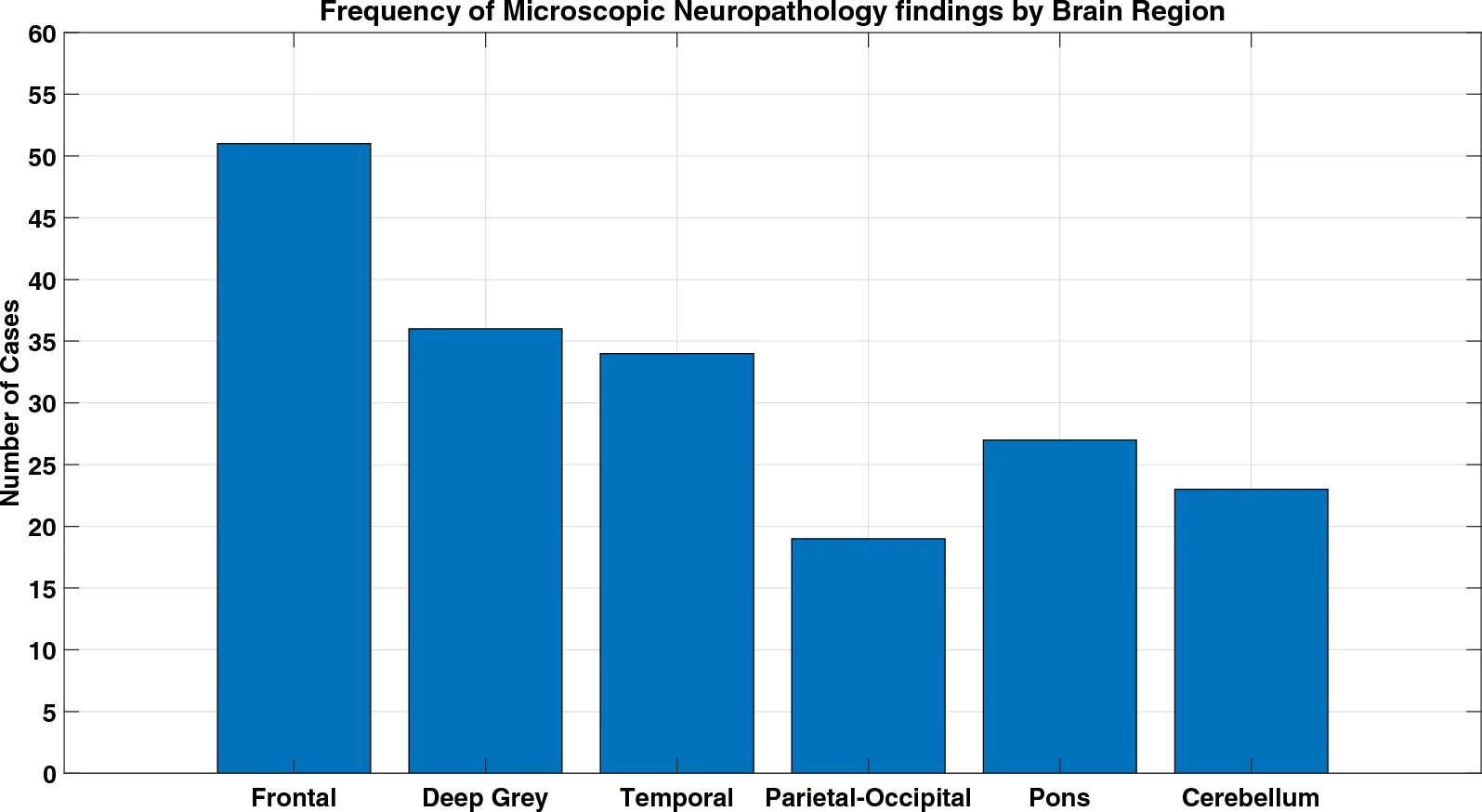
Frequency of microscopic neuropathology findings by brain region

Neuropathology revealed diverse injury patterns involving multiple brain regions. Frontal lobes showed gliosis, red neurons, and infarcts in most cases. Deep grey matter demonstrated hypoxic changes such as reactive gliosis and edema. Temporal lobes, particularly hippocampi, displayed ischemic necrosis. Parieto-occipital regions showed selective white matter changes. Pons and cerebellum occasionally exhibited infarcts, inflammatory injury, and Purkinje cell necrosis. Microscopy provided greater detail than gross pathology, often revealing injuries invisible macroscopically (Fig. 1).

### Region-Specific Neuropathological Patterns Across ECMO Modalities and Age Groups

**Fig. 2:**
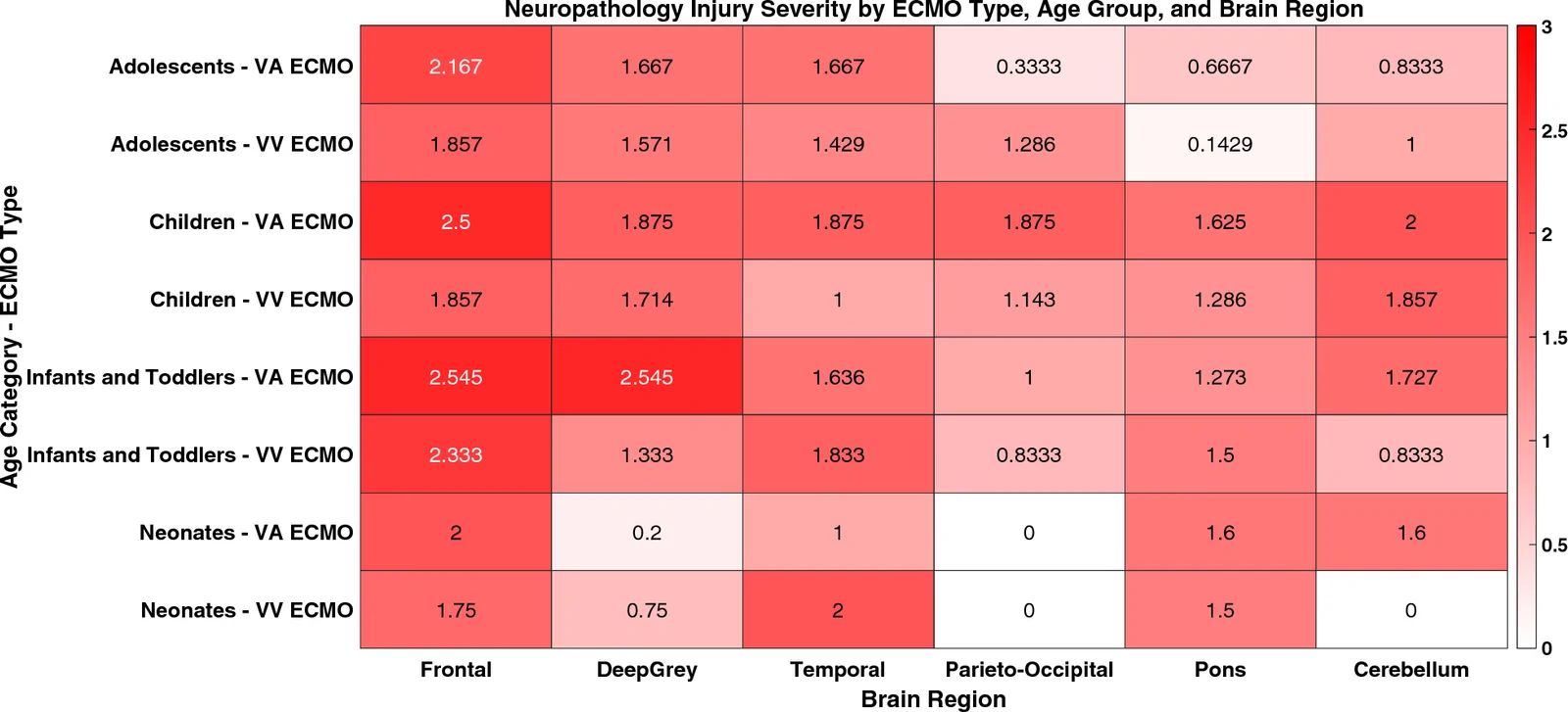
Heatmap of neuropathological injury severity by brain region, ECMO modality, and age

Neuropathologic patterns varied by age and ECMO modality **(**Fig. 2; ESM Table S2). In adolescents, VA ECMO was associated with moderate-to-severe injury in frontal, deep grey, and temporal regions, with milder pontine and cerebellar involvement. Cases supported with VV ECMO showed a similar but more regionally limited distribution of injury. In children, VA ECMO cases exhibited widespread severe injury, whereas VV ECMO cases demonstrated milder but still diffuse abnormalities. Infants and toddlers had severe frontal and deep grey matter injuries associated with both modalities, with additional moderate injuries in temporal, parieto-occipital, pontine, and cerebellar regions. Neonates showed moderate injury in frontal, temporal, and infratentorial regions for both VA and VV ECMO, although VV ECMO cases showed less frequent involvement of the cerebellum and parieto-occipital lobes. Cerebellar involvement overall was uncommon, with the exception of VA ECMO-supported neonates and children.

### Correlation between NIS and Gross Neuropathology Findings

Of the 54 pediatric decedents of ECMO with available neuropathologic data, 26 met inclusion criteria for the imaging–pathology correlation analysis. A total of 15 patients (58%) were classified as having severe injury, 3 (12%) as moderate, and 8 (31%) as mild. Gross neuropathologic findings correlated with these categories **(**ESM Table S3). Cases with severe NIS typically exhibited extensive or destructive patterns, including diffuse hypoxic-ischemic encephalopathy, organizing infarcts, and petechial hemorrhages. Moderate NIS cases showed more localized pathology, such as ischemic changes or non-necrotizing meningoencephalitis. In contrast, mild NIS cases often demonstrated either subtle or no grossly appreciable brain injury. This distribution supports a strong correlation between higher NIS and the extent of gross pathological brain injury.

### Total NIS Versus Global Injury Severity

**Fig. 3:**
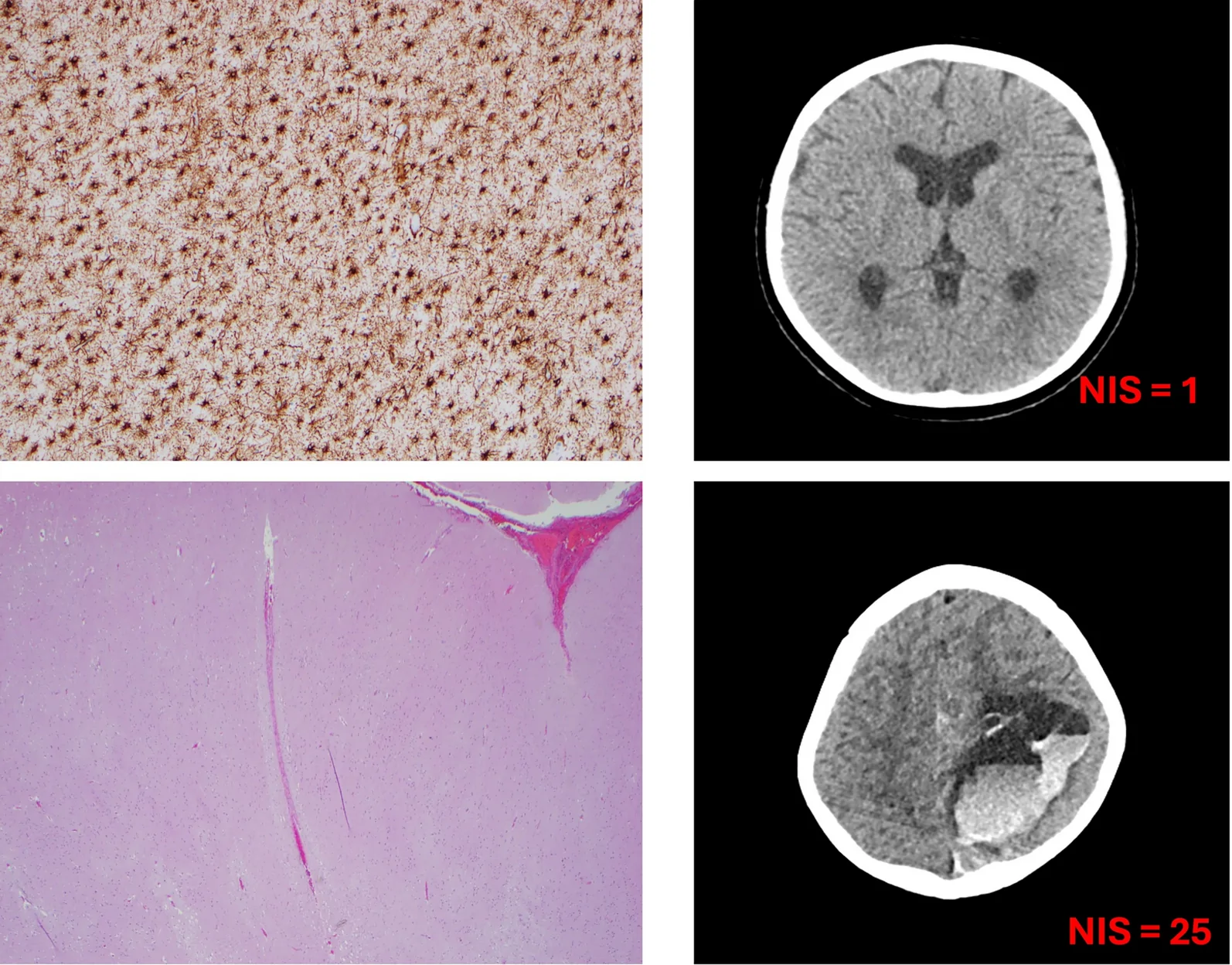
Histopathological and neuroimaging findings corresponding to Neurologic Injury Severity (NIS) scores. Top panel: NIS 1 (mild injury) characterized by mild patchy reactive gliosis in grey and white matter (left) and mild volume loss on imaging (right). Bottom panel: NIS 25 (severe injury) subarachnoid hemorrhage with ischemic parenchymal injury (left), with imaging evidence of large intracerebral, intraventricular, and extra-axial hemorrhages (right)

**Fig. 4:**
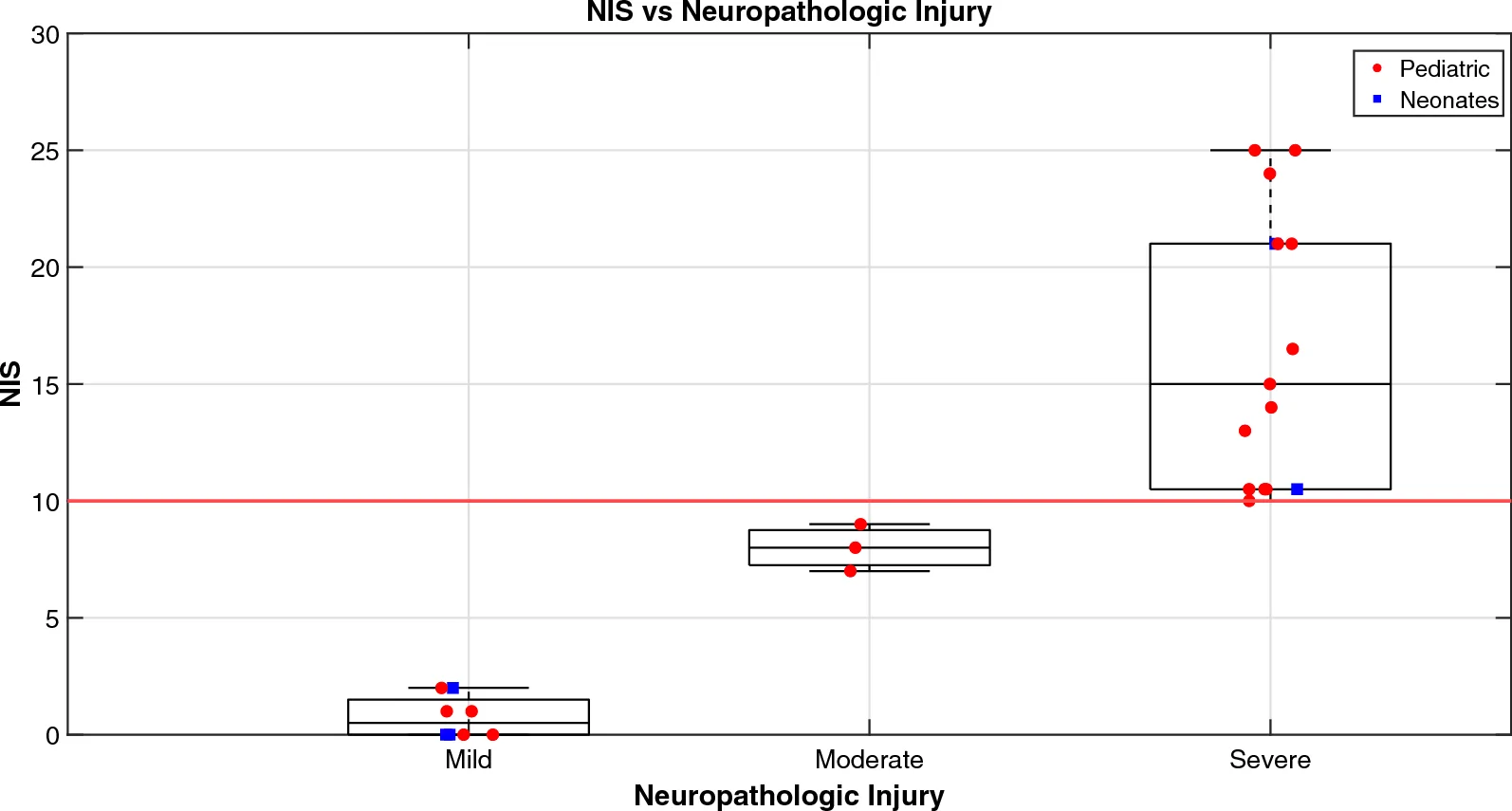
Box plot comparing total Neurologic Injury Severity (NIS) scores in patients stratified by overall neuropathologic injury classification (mild, moderate, severe)

Patients in the severe injury category demonstrated higher NIS scores than those in the mild and moderate categories (Fig. 3), reflecting greater radiologic injury burden. Patients classified as mild or moderate categories had scores within the predefined ranges corresponding to those categories. Figure 4 illustrates the distribution of total NIS scores across the three neuropathologic injury categories: mild, moderate, and severe.

### Region-Specific Correlations

**Fig. 5:**
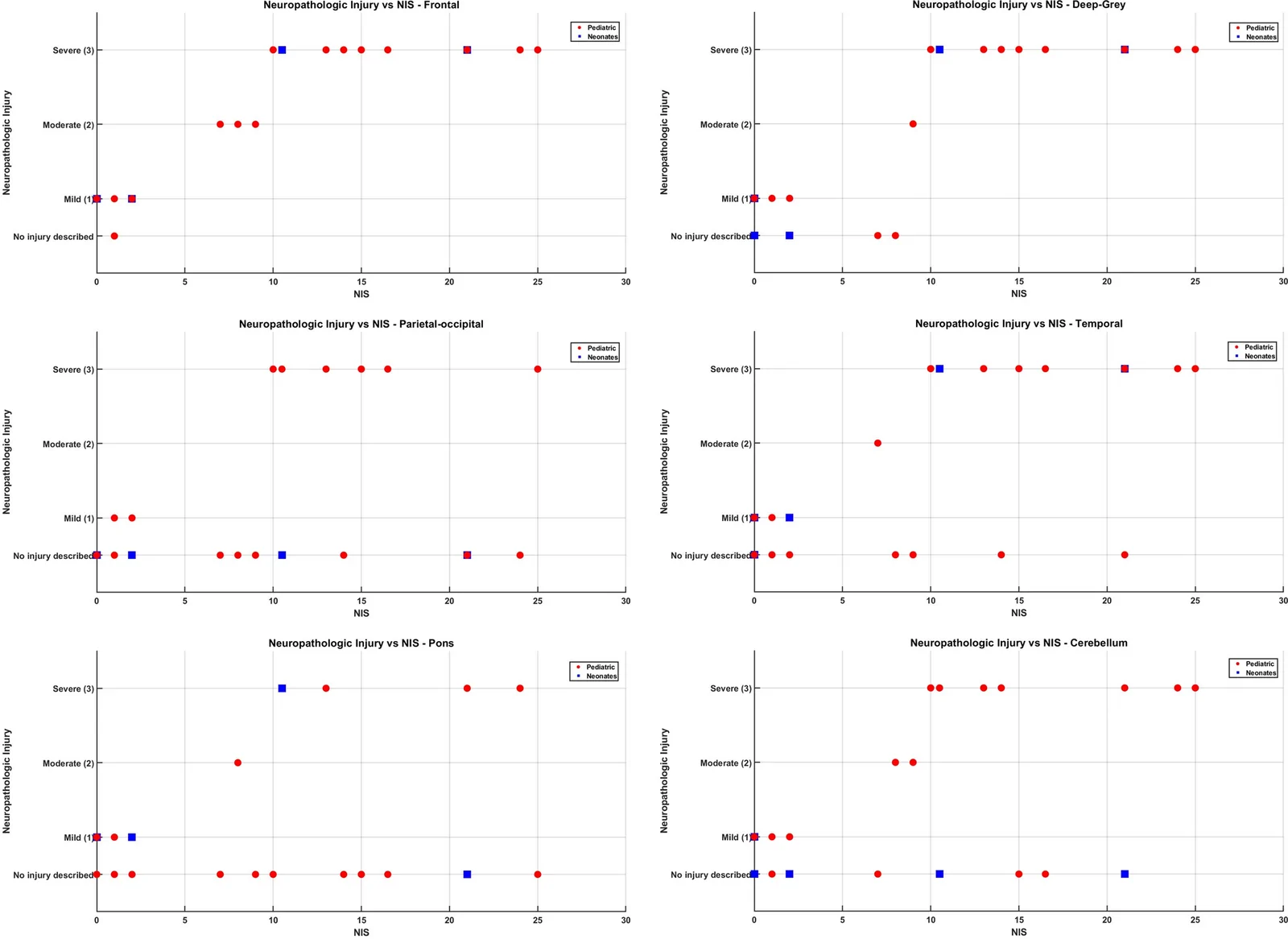
Region-specific correlation between neuropathologic injury level and Neurologic Injury Severity (NIS) score. Neonates are indicated with blue squares, while the remaining pediatric population is represented by black dots

Visual inspection of region-specific scatter plots (Fig. 5a–f), along with stratified analysis by injury level and age category, revealed consistent trends linking increasing histopathologic injury severity with higher Neurologic Injury Severity (NIS) scores across all six brain regions in a cohort of 26 patients. These trends were particularly robust in older pediatric groups. The enhanced sensitivity of CT imaging to deeper brain structures likely contributed to stronger correlations, particularly in the deep grey matter and posterior regions. Age played a critical role in both the pattern of injury and the alignment between imaging and pathology.

- Frontal lobes (Fig. 5a): severe injury (grade 3) was observed in 15 of 26 patients, predominantly infants and toddlers (*n* = 9), with additional cases in children (*n* = 2), adolescents (*n* = 2), and neonates (*n* = 2). Mild injury (grade 1) was present in seven patients, including three neonates, two infants and toddlers, one child and one adolescent. Patients with severe frontal injury exhibited NIS scores ≥ 10. Strong imaging–pathology concordance was most evident in infants and toddlers.
- Deep grey matter (Fig. 5b): severe injury was identified in 15 patients, including 9 infants and toddlers, 2 children, 2 adolescents, and 2 neonates. Mild injury occurred in six patients (including one neonate), while moderate injury was observed in one infant. This region showed one of the strongest correlations between injury level and NIS, likely due to CT’s enhanced sensitivity for deep structures.
- Parieto-occipital lobes (Fig. 5c): severe injury in the parieto-occipital region was identified in seven patients, predominantly among infants and toddlers (*n* = 5), with one case each in children and adolescents. Interestingly, two infants and toddlers and one child exhibited moderate NIS scores despite the absence of detectable parieto-occipital injury on standard H&E histology. In one infant, however, CD68 and GFAP immunostaining (applied to a subset of 19 patients) revealed occasionally activated microglia and reactive gliosis in the white matter, suggesting a subtle parieto-occipital injury not captured by conventional histological methods. For the remaining patients with cases with moderate NIS scores and no parieto-occipital injury, the neurological impairment is likely attributable to damage in other regions, specifically the frontal and/or deep grey matter, which can significantly impact function even when the parieto-occipital lobes appear unaffected. Notably, no moderate neuropathological injuries were recorded in this region.
- Temporal lobes (Fig. 5d): 13 patients had severe injury (8 infants and toddlers, 2 children, 2 adolescents, 1 neonate). Mild injury was seen in four patients, including two neonates, and moderate injury was seen in one infant. A stepwise increase in NIS scores paralleled injury severity, demonstrating strong imaging–pathology agreement across age groups.
- Pons (Fig. 5e): severe pontine injury was observed in eight patients, including five infants and toddlers, and one subject each from the adolescent, child, and neonate groups. One child exhibited moderate injury, while five patients, three of whom were neonates, had mild injury. Notably, seven patients (four infants and toddlers, and one each from the adolescent, child, and neonate groups) demonstrated severe NIS scores despite no described pontine injury on standard histopathology. Among these, five showed increased perivascular macrophages and reactive gliosis on GFAP and CD68 immunostaining, suggesting subtle or diffuse injury not captured by routine methods. Additionally, two infants with no described pontine injury had moderate NIS scores; both exhibited rare, activated microglia and mild reactive gliosis. Two other infants with no reported pontine injury were classified as having mild injury; one of them showed increased activated microglia in the white matter and mild reactive gliosis on GFAP and CD68. These findings highlight the potential for immunohistochemical markers to reveal underlying pathology not evident on conventional staining.
- Cerebellum (Fig. 5f): severe cerebellar injury was identified in ten patients, including seven infants and toddlers, two children, and one adolescent, all of whom had severe NIS scores. Moderate cerebellar injury was observed in one child and one infant, both corresponding with moderate NIS scores. Among five patients with mild cerebellar injury, only two had mild NIS scores, while the remaining three had NIS scores of zero. Of these three, two showed mild reactive gliosis in the white matter, suggesting subtle pathology not captured by standard scoring. Additionally, nine patients exhibited no histologic evidence of cerebellar injury despite having NIS scores ranging from mild to severe. In these cases, neurological impairment is likely attributable to injuries in other brain regions.

## Discussion

A strong correlation was observed between elevated NIS scores and severe histopathologic injury, both globally and regionally, reinforcing the clinical utility of the NIS framework for assessing neurologic burden during ECMO support. These findings suggest that structured imaging-based scoring, using modalities such as CT or head ultrasound, can serve as a meaningful surrogate for underlying neuropathology, particularly in settings where MRI is contraindicated or unavailable. The most robust imaging–pathology concordance was found in the frontal lobes, deep grey matter, and temporal lobes, where patients with severe injury consistently had NIS scores ≥ 10. However, the inclusion of neonates through adolescents introduces substantial developmental heterogeneity in cerebral vulnerability, perfusion physiology, and injury patterns. Although age-stratified analyses partially mitigate this limitation, the findings should not be extrapolated uniformly across developmental stages.

These regions may be particularly vulnerable to hypoxic-ischemic injury in pediatric patients with ECMO, aligning with prior reports of frontotemporal susceptibility. In contrast, the parieto-occipital lobes, pons, and cerebellum showed more frequent discordance, with several patients exhibiting elevated NIS scores despite no histopathologic injury described on standard H&E staining. Large focal vascular lesions were readily identified by neuroimaging; however, the clinically relevant uncertainty lies in the detection of diffuse hypoxic-ischemic or microscopic injury, which in some cases was evident only on histopathology or immunohistochemistry. These observations are further informed by the histopathologic findings, which provide important context for interpreting regional imaging–pathologic concordance.

Across all brain regions examined, the designation “no injury described” should not be interpreted as indicative of normal histology, particularly when relying solely on standard H&E staining. Subtle microscopic changes may go undetected, and in many cases, neurological impairment is attributable to injury in regions other than the one being assessed. Immunohistochemical markers such as CD68 and GFAP proved valuable in revealing reactive gliosis and microglial activation, offering insight into underlying pathology not captured by conventional methods. Overall, the analysis demonstrated a clear and consistent relationship between increasing histopathologic injury severity and elevated Neurologic Injury Severity (NIS) scores across all six brain regions. This correlation was especially strong in the frontal lobes, deep grey matter, and temporal regions, where CT imaging’s sensitivity to deeper structures likely enhanced detection and alignment with pathology. The trends were most robust in infants, toddlers, and older children, underscoring the reliability of NIS scoring in these age groups. In contrast, findings in neonates were more variable, highlighting the need for refined imaging protocols and further histopathologic validation to optimize NIS as a neuromonitoring tool during ECMO.

These findings support further refinement of both imaging protocols and histopathologic validation strategies to enhance the accuracy and reliability of NIS as a neuromonitoring biomarker during ECMO. Additionally, the variability in NIS scores among patients with severe pathology underscores the heterogeneity and multifactorial nature of brain injury in this population. The observed injuries cannot be attributed specifically to ECMO itself, as hypoxic-ischemic and hemorrhagic injury may arise from antecedent cardiac arrest, systemic hypoperfusion, or primary disease processes. In this context, ECMO likely serves as a marker of critical illness severity rather than the primary catalyst for injury, particularly for the 39% of patients requiring ECPR. The high incidence of deep grey matter and frontal lobe injury in our cohort likely reflects the cumulative impact of the pre-cannulation hypoxic-ischemic insult (median 34 min) and subsequent physiological instability during extracorporeal support. Similarly, differences observed between VV and VA ECMO likely reflect underlying indication and arrest physiology rather than ECMO modality alone. Contributing mechanisms may include altered cerebral autoregulation, fluctuating perfusion pressures, embolic events, and systemic inflammatory responses, particularly in venoarterial ECMO configurations [25, 26].

Emerging studies employing plasma biomarkers and machine learning have further highlighted this complexity by identifying dynamic injury patterns that evolve throughout ECMO support. For instance, machine learning models have been developed to predict brain injury in pediatric patients with ECMO, demonstrating the potential of these tools in enhancing diagnostic accuracy and patient management [27–29].

Our study reinforces the utility of neuroimaging, particularly CT, in detecting significant neurologic injury in pediatric patients with ECMO. While MRI is often considered the gold standard for identifying white matter injuries and microhemorrhages due to its superior soft tissue contrast, its application is frequently limited in ECMO settings because of equipment incompatibilities and patient instability [30]. CT, in contrast, is more accessible and faster, making it a practical choice in critical care scenarios. Notably, our findings align with previous research indicating that CT has a high positive predictive value for detecting severe brain injuries when performed within 14 days of ECMO initiation [31]. It is important to recognize that, while MRI offers higher soft tissue resolution, CT provides sufficient detail to identify clinically significant injuries, especially in acute settings. Therefore, CT serves as a reliable diagnostic tool for early detection of severe neurologic injuries in this patient population.

Furthermore, adjunctive neuromonitoring modalities, including amplitude-integrated electroencephalography (aEEG) [32], commercial cerebral oximeters [33], and diffuse optical spectroscopies [34, 35], have gained traction for their ability to provide real-time cerebral monitoring in patients with ECMO. Their incorporation into clinical protocols may enhance the accuracy and timeliness of neurologic assessments during ECMO support. For example, a study by Sanford et al. demonstrated that disruption of cerebral autoregulation, as measured by diffuse correlation spectroscopy, was associated with higher radiographic neurologic injury scores in pediatric patients with ECMO [34].

This study has several limitations. This was a retrospective, single-center, retrospective study with a small overall (*n* = 54) and smaller imaging–pathology cohort (*n* = 26), which limits statistical power and generalizability. Neuroimaging was obtained up to 7 days prior to death; therefore, additional cerebral injury may have occurred between imaging acquisition and neuropathologic examination, potentially contributing to imaging–pathology discordance. Imaging modalities varied across patients, and CT and head ultrasound lack sensitivity for detecting diffuse, early, or microscopic injury. Although standardized histological sampling was employed, focal pathology may have been missed. Additionally, early ischemic injury that could be characterized by immunostains such as amyloid precursor protein (APP) were not performed in this study.

Furthermore, combining patients supported with veno-venous ECMO for respiratory failure and veno-arterial ECMO for extracorporeal cardiopulmonary resuscitation introduces substantial physiological heterogeneity and limits mechanistic inference regarding injury patterns. Finally, although imaging and histopathologic assessments were performed in a blinded manner, interobserver variability cannot be excluded; therefore, these findings should be considered exploratory and hypothesis-generating.

## Conclusions

Neuroimaging, particularly CT interpreted through structured severity scoring, correlates strongly with neuropathologic findings in pediatric decedents of ECMO. Frontal and deep grey regions demonstrated the clearest alignment, while regional mismatches underscore the need for enhanced histologic methods and adjunctive monitoring. Prospective multicenter studies with standardized imaging protocols and biomarker validation are essential to advance neurologic surveillance in this population.

## Supplementary Information

Below is the link to the electronic supplementary material.

Supplementary file1 (PDF 105 KB)
