# Supplementary material for: Neuroimaging and Neuropathologic Findings in Pediatric Patients with ECMO: An Imaging–Pathology Comparison
Source: Neurocrit Care. 2026 May 26;45(2):787–97. doi: 10.1007/s12028-026-02545-9 (PMC13642489; doi:10.1007/s12028-026-02545-9)
Supplement: Supplementary file 1 [file 12028_2026_2545_MOESM1_ESM.pdf]

Supplemental digital content

Injury Level	Histopathologic Criteria	Regions Assessed	Interpretative Features
**Mild**	Nonspecific or limited findings:<br>• Disorganized myelination<br>• Absence of white matter injury<br>• Slight ventricular dilation	• Frontal cortex<br>• Deep grey matter<br>• Temporal<br>• Parieto-occipital<br>• Pons<br>• Cerebellum	Findings are subtle, often isolated, and not associated with overt structural damage
**Moderate**	Focal abnormalities:<br>• White matter pallor<br>• White matter edema<br>• Focal hemorrhage<br>• Focal ischemic injury<br>• Red neurons alone<br>• Neuronal necrosis without white matter involvement	• Frontal cortex<br>• Deep grey matter<br>• Temporal<br>• Parieto-occipital<br>• Pons<br>• Cerebellum	Injury involves localized structural change without diffuse or multifocal damage
**Severe**	Widespread or multifocal damage:<br>• White matter gliosis<br>• Subdural hemorrhage with neuronal necrosis<br>• Extensive necrosis<br>• Acute and organizing infarct<br>• Necrotic hemorrhage<br>• Diffuse ischemic injury<br>• Neuronal dropout	• Frontal cortex<br>• Deep grey matter<br>• Temporal<br>• Parieto-occipital<br>• Pons<br>• Cerebellum	Indicates irreversible injury affecting multiple regions and both grey and white matter

Table 1: Tiered Classification of Neuropathological Injury

Age Category	ECMO Type	Frontal	Deep Grey	Temporal	Parieto-Occipital	Pons	Cerebellum	Subjects Count
**Adolescents**	VA ECMO	Severe	Moderate	Moderate	Mild	Mild	Mild	**6**
**Adolescents**	VV ECMO	Moderate	Moderate	Moderate	Moderate	Mild	Mild	**7**
**Children**	VA ECMO	Severe	Severe	Severe	Severe	Moderate	Severe	**8**
**Children**	VV ECMO	Moderate	Moderate	Mild	Moderate	Moderate	Moderate	**7**
**Infants and Toddlers**	VA ECMO	Severe	Severe	Moderate	Mild	Moderate	Moderate	**11**
**Infants and Toddlers**	VV ECMO	Severe	Moderate	Moderate	Mild	Moderate	Mild	**6**
**Neonates**	VA ECMO	Moderate	Mild	Mild	None	Moderate	Moderate	**5**
**Neonates**	VV ECMO	Moderate	Mild	Moderate	None	Moderate	None	**4**

Table 2: Most Common Neuropathological Injury Levels by Age, ECMO Modality, and Brain Region.

Injury Severity	NIS Range	Number of Subjects	Gross Pathology Findings
**Mild**	0-3	8 (31%)	No visible infarcts, subtle ischemic changes, or normal gross appearance
**Moderate**	4-9	3 (12%)	Focal ischemia, localized infarcts, or non-necrotizing inflammation
**Severe**	≥ 10	15 (58%)	Diffuse HIE, organizing infarcts, petechial hemorrhages, widespread edema

Table 3. Correlation between NIS and Gross Neuropathology
